# Ovarian Ectopic Pregnancy in a Female With Mitral Valve Repair and Mitral Stenosis: A Case Report on a Rare Type of Ectopic Pregnancy

**DOI:** 10.7759/cureus.68112

**Published:** 2024-08-29

**Authors:** Sukanya Singh, Surekha Tayade, Drashti Patel

**Affiliations:** 1 Department of Obstetrics and Gynecology, Jawaharlal Nehru Medical College, Datta Meghe Institute of Medical Sciences, Wardha, IND

**Keywords:** warfarin, rhd pregnancy, ovarian ectopic pregnancy, mitral valve replacement, chronic anticoagulants

## Abstract

Ovarian ectopic pregnancy (OEP) occurs in cases where the fertilized egg is implanted outside the uterus in either of the ovaries. Assisted reproductive technologies and intrauterine device failure are high-risk factors associated with ovarian ectopic pregnancy. Pregnancies categorized under OEP have a higher risk of serious morbidities to maternal health. Clinical presentations of OEP are usually noted as abdominal pain and vaginal bleeding. Transvaginal ultrasound is considered the preferred primary modality for the diagnosis of OEC. It can be life threatening, especially in patients with mitral valve replacement (MVR) or heart diseases like rheumatic heart disease, majorly due to anticoagulant therapy. Pregnancy in MVR-mitral stenosis patients has been reported to have an increased risk of obstetric hemorrhage, miscarriage, and associated complications during delivery. Management of OEP depends on the patient’s physical and clinical condition, with a primary focus on preserving the affected ovary function. This is a case of a 35-year-old pregnant female with a history of MVR presented with per vaginal bleeding and ruptured ectopic pregnancy. Radio imaging showed the product of conception attached to the right ovarian cyst. The patient was counseled for exploratory laparotomy and subsequently had right ovarian cystectomy alone with bilateral tubal ligation by modified Pomeroy’s method.

## Introduction

Ovarian ectopic pregnancy (OEP) is a rare type of ectopic pregnancy. The reported incidence of OEP is between 1/7,000 and 1/40,000 live births and 0.5%-3% of all ectopic gestations. Major underlying causes reported for OEP are the use of intrauterine devices and ovulatory drugs and increased use of assisted reproductive techniques (ARTs), given increased infertility rates [1,2]. Also, research literature attributes increased incidence to the availability of better diagnostic tools. However, there are high chances of OEP being misdiagnosed as hemorrhagic corpus luteum or ruptured tubal ectopic pregnancy and its interventional management [3]. Imaging modalities such as high-resolution transvaginal ultrasonography can be a simple yet effective tool for diagnosing OEP, but it requires a certain level of experience. These pregnancies carry a significant risk to maternal health and are often associated with hemorrhage, uterine rupture if not timely diagnosed, and emergency ovary removal. This is further complicated in patients who underwent mitral valve replacement (MVR) or are affected by heart diseases such as rheumatic heart disease (RHD) [4]. Associated risks in these patients have also been studied based on the type of valve and anticoagulant therapy used [5]. In this case report, we present a 35-year-old female with a history of MVR and RHD with ectopic ovarian pregnancy and its management.

## Case presentation

**Figure 1:**
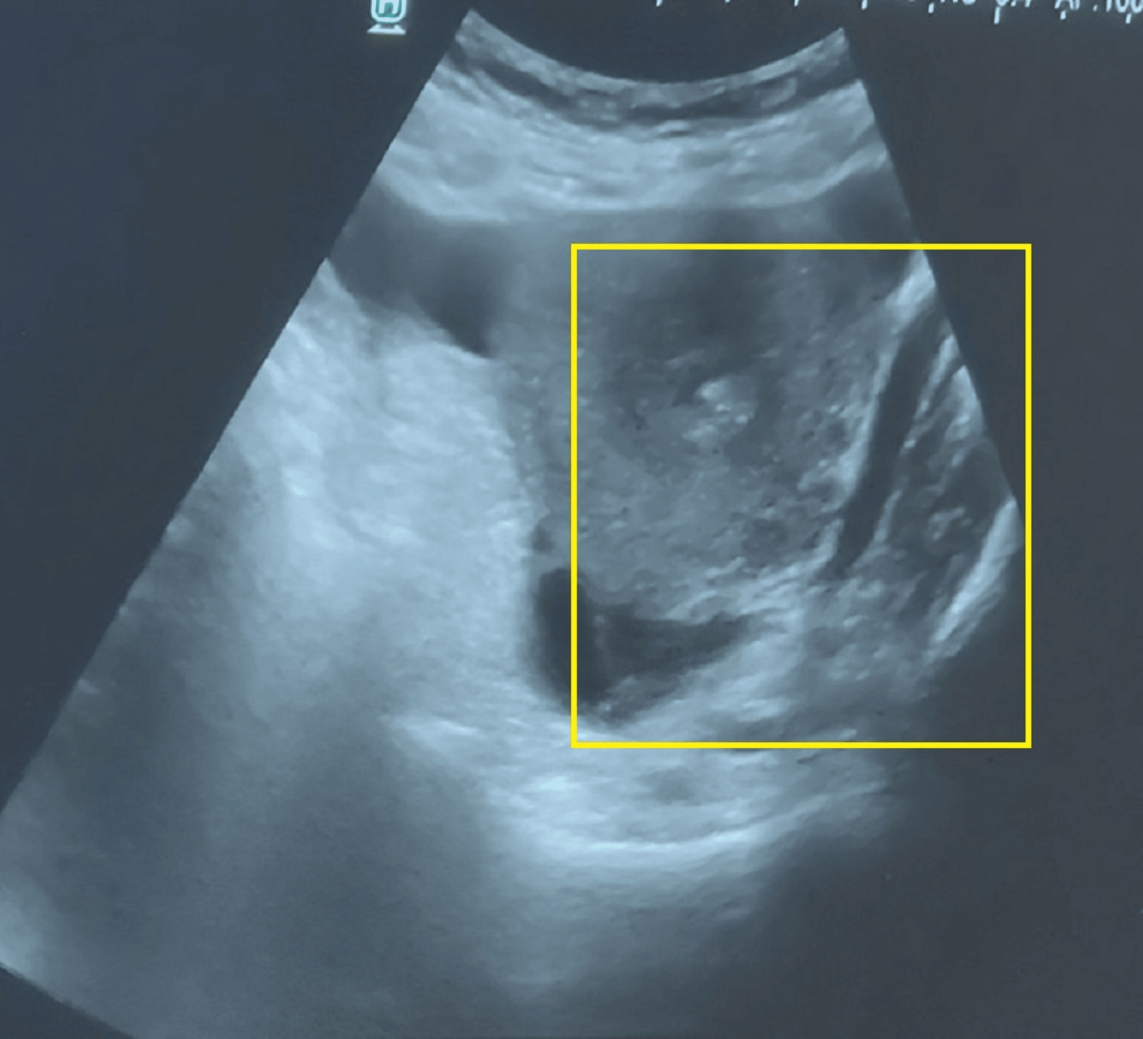
USG image of the patient USG: ultrasound

A 35-year-old female gravida 3, para 2 and two live issues with mitral valve repair and a known history of RHD with mitral stenosis (MS) and severe anemia presented at our hospital with a complaint of sudden-onset abdominal pain that has gradually worsened over the past 15 days. The pain had no aggravation or relieving factors. She also complained of per vaginal bleeding for 15 days, vomiting and nausea for two days, and a high-grade fever of 103°. She was referred to Acharya Vinoba Bhave Rural Hospital, Maharashtra, India, for further management of the possibility of ruptured ectopic pregnancy. The patient underwent cardiac surgery 13 years ago, but there are no available documents. The patient has been taking a 5-mg dose of warfarin once daily for the past 13 years. She had no medical history of diabetes, hypertension, bronchial asthma, or epilepsy. Her last childbirth was 18 years ago. Both children were delivered at full term by normal vaginal delivery. On arrival, the patient had an international normalized ratio (INR) of 10.6 and was afebrile, and further details regarding the vitals were provided. The patient's blood pressure was measured at 120/80 mmHg with a heart rate of 96 beats per minute. Pallor was noted, while edema was absent. Both cardiovascular and respiratory system examinations revealed normal findings. Upon abdominal examination, the patient's abdomen exhibited a scaphoid shape and moved with respiration, and the umbilicus was centrally positioned and of normal shape. On deep palpation, a mass of approximately 3 x 3 cm in the iliac region was felt. During per vaginal examination, it was found that the uterus was bulky, measuring 8-10 weeks in size. Both the fornices were free with no cervical motion tenderness. Her serum beta-human chorionic gonadotropin (HCG) levels were determined to be 2105 milli-international units per milliliter (mIU/mL). A provisional diagnosis was made based on clinical examination, transabdominal ultrasound (USG) imaging, and laboratory reports for a ruptured ectopic pregnancy. A decision for exploratory laparotomy was then made (Figure 1).

**Figure 2:**
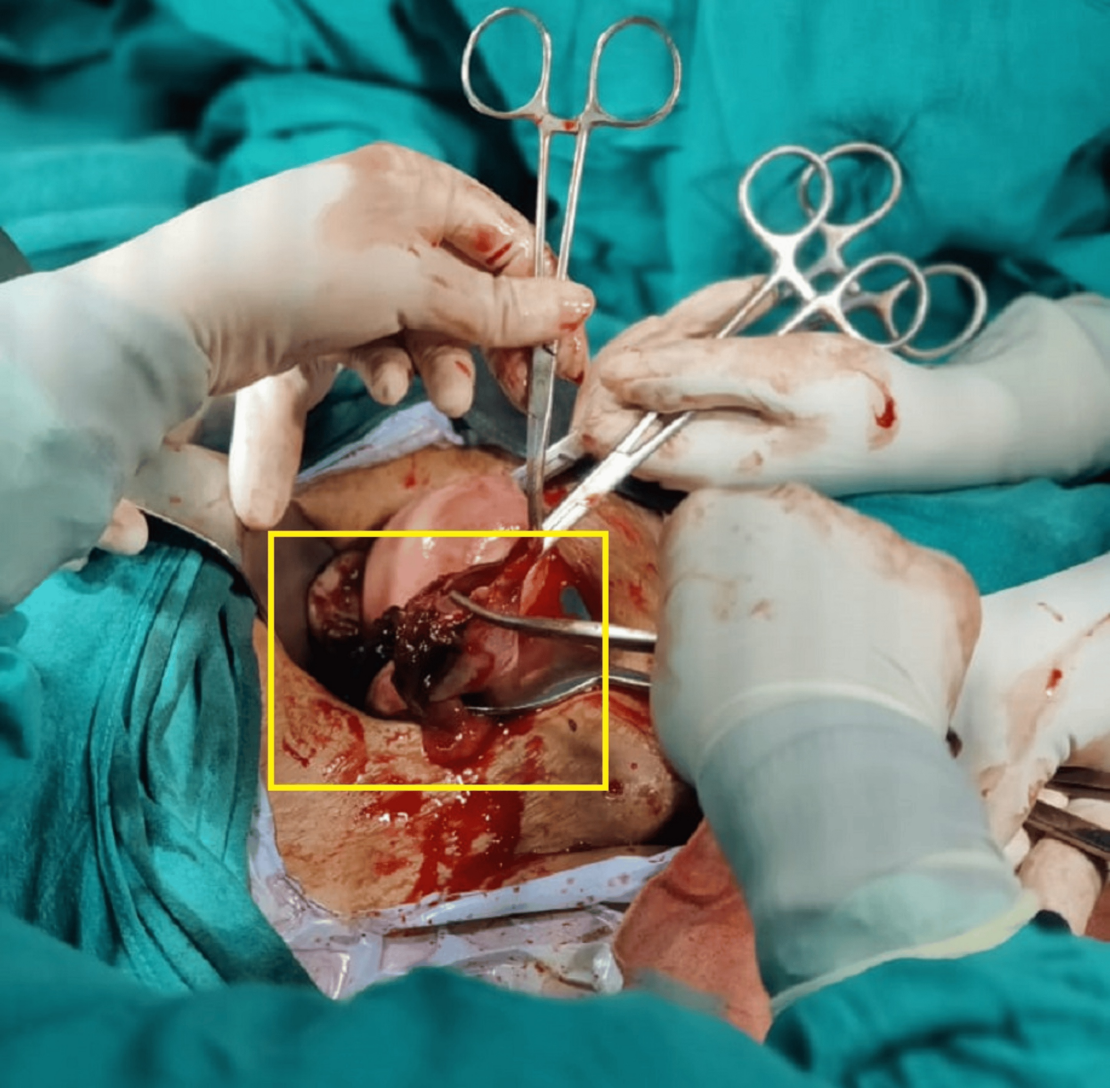
Intraoperative image of the patient

**Figure 3:**
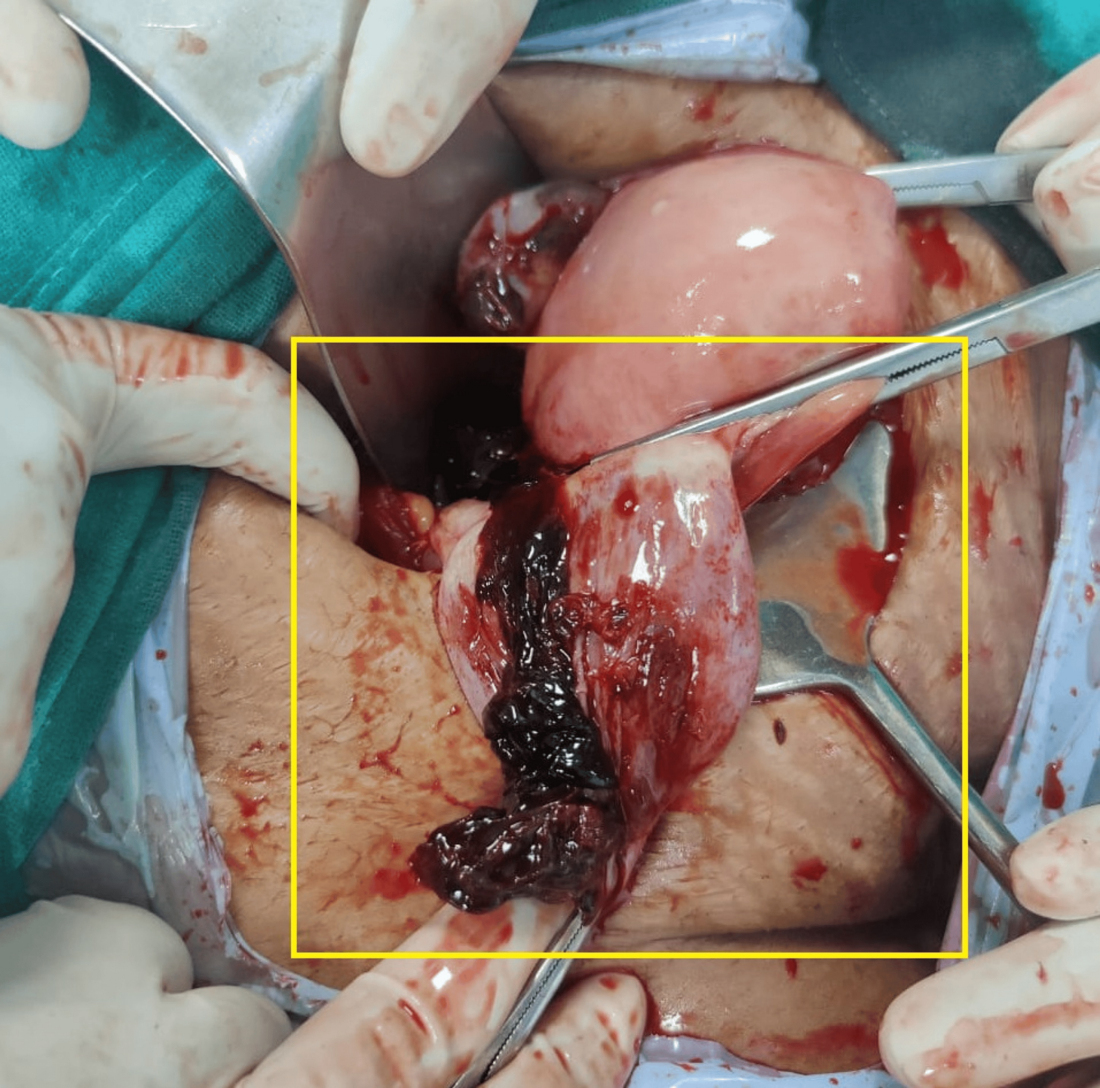
Intraoperative image of the patient revealing right-sided ruptured OEP OEP: ovarian ectopic pregnancy

**Figure 4:**
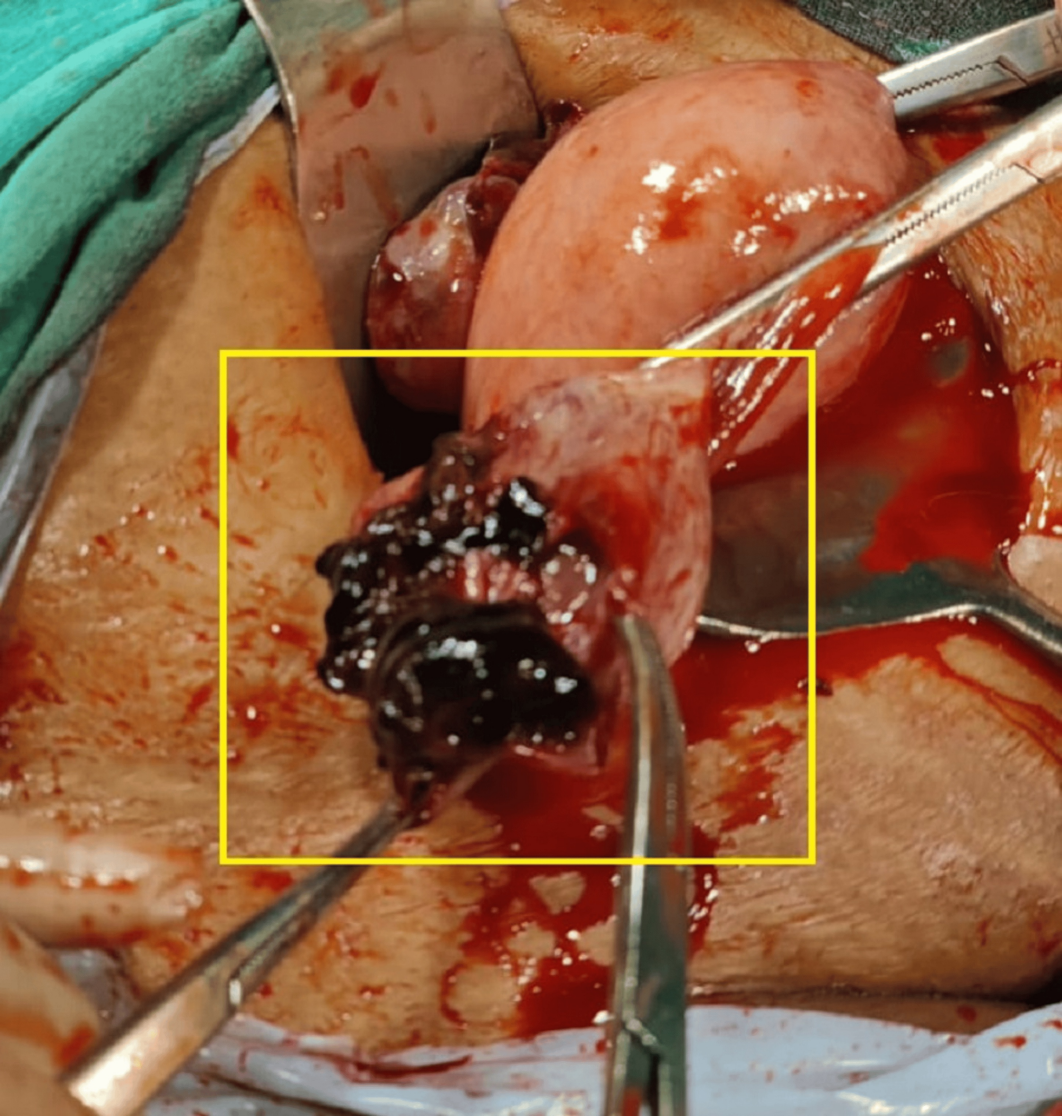
Right-sided ruptured ovarian ectopic pregnancy seen with the product of conception attached to the ovarian cyst wall

The patient underwent a two-dimensional (2D) echocardiogram, revealing a dilated left atrium/left ventricle with severe mitral regurgitation (MR), mild MS, and severe aortic regurgitation (AR). The mitral valve annulus was 36 mm. Mild tricuspid regurgitation (TR) was observed in conjunction with a right ventricular systolic pressure (RVSP) of 26 mmHg, alongside good biventricular function, and a left ventricular ejection fraction of 60%. High-risk consent was obtained. The patient received general anesthesia, and an approximately 8-cm incision was made to open the abdomen in layers with appropriate precautions. The peritoneal cavity was accessed, and a positive hemoperitoneum of approximately 150 cc was detected. A right-sided ruptured ovarian ectopic pregnancy was observed, with the product of conception attached to the ovarian cyst wall. The product of conception was removed, and cystectomy was performed. Bilateral tubal ligation was performed using the modified Pomeroy's method and sent for histopathological examination. Blood clots of approximately 3 x 3 cm were observed with no active bleeding (Figures 2-4).

A peritoneal wash was carried out, hemostasis was achieved, the abdomen was closed in layers, and the dressing was done without any postoperative events. The patient underwent a follow-up examination seven days postoperatively. Serum beta-HCG levels were repeated after 24 and 48 hours with results of 1,490 and 234 mIU/mL, respectively. On postoperative day 4, a 2D echocardiography revealed that the prosthetic metallic valve was in place with trivial MR. The gradient across the mitral valve was measured at 8/3 mmHg with a mitral valve area of 2.08 cm2 as determined by pressure half-time. Trivial TR was observed, with an RVSP of 20 mmHg. Additionally, there was no AR, the inferior vena cava was normal and collapsed with respiration, and there were no signs of pulmonary embolism or infective endocarditis. The biventricular systolic function was found to be normal. The USG report on day 4 showed hepatomegaly and no free fluid in the peritoneal cavity, with 4 mm endometrial thickness and uterus dimensions of 8.2 x 4.1 x 3.2 cm. INR was repeated serially. Under a cardiologist's supervision, the warfarin dose was tapered (Table 1).

**Table 1:** INR readings of the patient with response to warfarin dosage APTT: activated partial thromboplastin time; INR: international normalized ratio

Postoperative day	APTT		Prothrombin time		INR	Warfarin dose (in mg)
	Patient	Control	Patient	Control		
4	54.7	29.5	67.9	11.9	6.33	5
5	39.0	29.5	29.8	11.9	2.64	5
6	40.3	29.5	34.0	11.9	3.04	3
7	33.4	29.5	23.4	11.9	2.05	3
8	33.8	29.5	18.6	11.9	1.60	2
9	32.2	29.5	18.2	11.9	1.52	2
10	30.1	29.5	17.2	11.9	1.47	2

**Figure 5:**
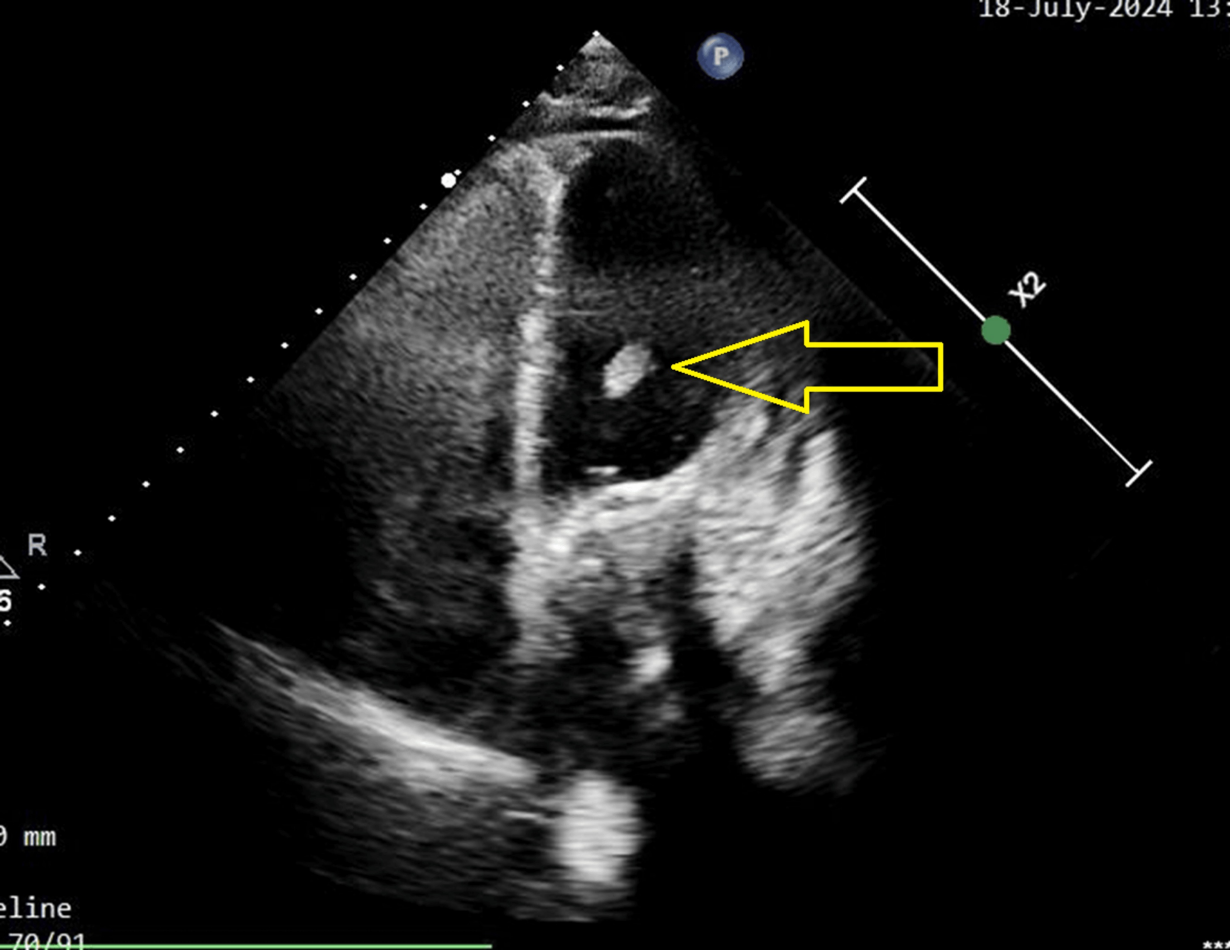
Postprocedure 2D echocardiography image showing MVR (yellow arrow) 2D: two dimensional; MVR: mitral valve replacement

The peripheral smear revealed normocytic, normochromic anemia with mild anisopoikilocytosis, which displayed a few pencil cells. The histopathology report of the specimen revealed products of conception. Postprocedure echocardiography was done (Figure 5).

A final diagnosis of ruptured OEP with a history of MVR-MS and RHD, along with an unspecified ectopic pregnancy, was made. The patient was counseled for an exploratory laparotomy to address the ruptured OEP.

## Discussion

The incidence of ectopic pregnancies has been reported to be between 1% and 2%, most of which are fallopian tube pregnancies. Ovary implantation of the fertilized egg has been observed in a range of one in 2,100-7,000 pregnancies, accounting for 3% of total ectopic pregnancies [6]. Though OEP is rare, increasing ART therapies for fertilization is increasing the chances of OEP. It has also been associated with an increased risk of hemorrhage and other associated comorbidities, which is further increased by anticoagulants, which are mandatory for patients undergoing MVR [4-6]. These anticoagulants have been reported to cause drug-induced coagulopathy. This, when related to reproductive health, has been linked to coagulopathy hemorrhage in young females. Patients on prolonged anticoagulants have been reported to have some or more incidences of bleeding, such as ovarian hemorrhage, intracranial hemorrhage, gum bleeding, urinary tract bleeding, and postmenopausal bleeding [7]. Females with MVR-MS and on anticoagulants are at high risk both during the pregnancy tenure and a few days postpartum. The use of anticoagulants has been reported to increase the risk of premature birth, fetal hemorrhage, renal and hepatic toxicities, and others [5]. Pregnancy and its associated morbidities in MVR-MS patients have also been associated with the type of valve being used, i.e., mechanical valve or bioprosthetic valve. Incidences of thromboembolism were found to vary from 4.8% to 7.3% in mitral valve location and from 0.3% to 0.7% in aortic position in research involving patients with prosthetic valves [8]. A systematic review assessing the risk of pregnancy post-MVR has reported an increased risk of obstetric hemorrhage during delivery. It was also noted that there is currently no safe and balanced regimen available that can simultaneously balance desired maternal and fetal outcomes [5]. A meta-analysis study focusing on a warfarin dose limited to 5 mg/day found that most fetal losses were due to spontaneous abortions, with an embryopathy rate of 0.9% and no maternal fatalities. The study also reported bleeding events, with thromboembolism at 3.4% and prosthetic valve thrombosis at 1.8% [9]. There are no case reports of patients with MVR-MS and OEP reported to date, which adds to the uniqueness of this case and its management. Due to the attributed high risk of OEP and an added risk of a history of MVR and anticoagulants, patient management becomes critical in terms of maintaining the balance between maternal and fetal health. This case was managed by a mini-laparotomy procedure, which was helpful in an uneventful recovery. Most of the modalities of OEP are managed depending on the patient's clinical condition [2,7-9]. Laparotomy, embolization, hemoperitoneum, or hysterectomy might be suggested majorly, based on with or without fertility preservation. Scar ectopic pregnancy type IIIb patients were at a high risk of intraoperative hemorrhage and were recommended to be managed by uterine artery embolization followed by laparoscopic excision, which was also carried out in our case.

## Conclusions

Females of reproductive age with MVR-MS and on anticoagulants are at high risk of hemorrhage and vaginal bleeding can be marked as an early sign to address the issue as an emergency, to avoid adverse outcomes. A similar comprehensive management approach is required in the management of ovarian ectopic pregnancies, especially in the first trimester as chances of uterine rupture and internal hemorrhage are high.
